# Rodent Ulcer and Its Treatment by Zinc Ions

**Published:** 1906-10-20

**Authors:** H. Lewis Jones

**Affiliations:** Med. Off. in charge of the Electrical Department of St. Bartholomew's Hospital


### Recent Clinical Methods.

### RODENT ULCER AND ITS TREATMENT BY ZINC IONS.

By H. Lewis Jones, M.D., M.A.Cantab., Med. Off. in charge of the Electrical Department of St. Bartholomew's Hospital.

(*Specially Reported for* The Hospital.)

At the Medical Graduates' College and Polyclinic on the 10th inst. the following interesting new method of treating rodent ulcer was demonstrated. The lecturer said rodent ulcer has been robbed of most of the difficulties of its treatment by the use of radium, which is a costly material, and by the *x*-rays, which require an elaborate apparatus and had also a small amount of risk attending their procedure. The treatment by zinc "ions" is simple and can be managed by anyone possessing a portable battery, and the results are equally good. The method is based upon the principle of the introduction of the "ions" of zinc into the tissues of the ulcer and the deposition of zinc in the protoplasm of its tissues, causing rodent ulcer which may have resisted ordinary treatment to assume the appearance of a simple sore, and to heal up in about ten days or a fortnight. A patient is here shown who suffered with rodent ulcer for ten years. She was sent to me by Mr. Bowlby and I made the application with zinc "ions" for ten or twelve minutes. Three weeks later the ulcer was completely healed and the point of ulceration looks now to be completely healthy and sound. The case represents the ordinary condition and the behaviour of rodent ulcer to the treatment. Most of my work has been with small rodents. In the large ones, with a definite thickness, there is more difficulty in obtaining good results.

### The History.

The real discoverer of this method was Dr. Stephane Leduc, Professor at the Medical School of Nantes in France, who published a case in the *Archives d'electricité Medicale* in 1903 (page 734), described as "The cicatrisation of a rodent ulcer in the angle of the nose of five years' standing after one application of electrosysis of zinc." A solution of zinc chloride (1 per cent.) on lint was applied at the positive pole. In December 1903, I happened to notice this paper, and in the following year had an opportunity of treating a case of rodent ulcer on the cheek in an elderly woman, using chloride of zinc at the positive pole of a battery, for 10 or 12 minutes. A fortnight later the ulcer had healed, and continued with no sign of recurrence during the past two years. I can now hardly see where the ulcer has been. I read a paper before the Hunterian Society recording seven successful cases so treated. I can now report eighteen cases, fifteen of which are absolute successes, and, therefore, I am justified in thinking that the method is really a first-rate one.

### The Physics of the Treatment.

It is necessary to define an "ion." In a solution of sulphate of zinc in water the zinc exists in an "ionic" state, that is to say each particle or molecule carries its appropriate positive charge of electricity. To return to the metallic state, it must be deprived of this charge. The difference between the "ion" and the ordinary zinc is the difference of its electric charge. In its solution the zinc exists in a partially dissociated form, and so with the sulphuric acid or other solvent. When electrical conduction takes place through such a solution, movement of material particles takes place, and the "ions" in the case of zinc move towards the negative pole. Conversely the sulphuric acid "ions"—$SO_4$—go to the positive pole. One makes use of these migrations of the ions to introduce them into the body. If at the positive pole you have a solution of zinc sulphate, directly the current begins to flow the zinc begins to move into the tissues, and to permeate the cells of the region at which the application is made. Rodent ulcer being generally superficial lends itself extremely well to medication of this kind. The discovery that zinc had a specific effect may have been accidental, but zinc in some form or other has, from its beneficial effects, long formed a favourite application to wounds, and its powers are brought out to the utmost when it is forced into the cells of the tissue. A zinc lotion, or ointment, gives extremely little penetration, and what little zinc is absorbed is probably carried off by the lymph stream and distributed over the system generally. Hence its effects are relatively feeble when used in this way. But when the "ion" is forced in electrically it enters not only into the lymph spaces, but into the cells themselves and becomes a part of the protoplasm itself, stays there and exercises its good effects much longer. The $SO_4$ ions migrate inwards from the body towards the positive pole, and play a minor rôle, but one not without use. As the conduction of electricity in any solution is only effected by the movement of the "ions," each carrying its charge of electricity—the exact amount of any "ion" introduced into the patient can be measured by the aid of the galvanometer. At an ordinary sitting the exact amount of zinc introduced is calculated to be two or three milligrams of zinc. But it is concentrated exactly at the place where it is wanted.

An experiment was made by placing two rabbits

ARMY MEDICAL LIBRARY

in electrical contact. Pads moistened with strychnine sulphate were applied to the bodies of both rabbits and a current passed. At the positive pole—strychnine having a tendency to go to the negative would move inwards to the negative pole—and, therefore, the rabbit at the positive pole was poisoned with strychnine. The animal at the negative pole showed no effects. One must learn in using electrical medication which "ions" move towards the positive pole and which towards the negative. Most metallic "ions" and all the alkaloids move from the positive to the negative pole, and must, therefore, be introduced at the positive pole. Acids, on the other hand, move in the other direction that is to say they are introduced at the negative pole.

### Practical Details.

The apparatus is extremely simple—a portable battery with wires—a pad attached to the negative pole and a disc or rod of zinc attached to the positive. The piece of zinc must be covered with two or three layers of lint wetted with a 4 per cent. solution of zinc sulphate. I usually keep a number of zinc electrodes of different sizes ready in a solution of sulphate of zinc. These should not be touched with the fingers, as sodium chloride and other impurities may be introduced. The zinc disc is held over the rodent ulcer, the circuit is closed and the current slowly turned on until a current of ten milliampères is passing. The application is continued for ten, twelve or fifteen minutes according to the thickness of the individual ulcer. Patients can bear up to ten milliampères without complaining. The application gives a burning sensation like a mustard plaster. In sensitive patients the application of pure cocaine applied on the pad may be used as a preliminary to make the part anæsthetic.

### Cases of Difficulty.

One learns from difficult cases that the condition of the surface of the ulcer is important in connection with the zinc treatment of rodent ulcer. Some are moist, some are dry, and others show small pearly modules round the margins; they vary a good deal in character. Crusts at the edge tend to make the zinc pass in unequally. One can remove the crusts beforehand, and so get a uniform penetration to make the centre heal as well as the margin. If the surface is wax-like the treatment can generally be accelerated by using a needle of zinc made by filing some zinc wire, and then puncturing the individual tubercules for one minute each as a preliminary with two milligrames of current. Then the main area may be attacked more satisfactorily. Cases which have been operated on and have relapsed are difficult because there are apt to be numerous unhealthy points, become by lapse of time thicker. When the ulcer extends for more than two millimetres in thickness longer applications are needed to cause the zinc to penetrate.

The lecturer detailed altogether eighteen cases, of which three were still under treatment, and, therefore, cannot be considered complete. He continued: I am sure that if any of you try this method you will get really good results. It is not only in my hands that this method has been a success. In the *Medical Electrology and Radiology*, of August last, Dr. David Arthur reports four cases—two healed in fourteen days after one application, a third after two applications, and the remaining case, although it was described by the author as having looked red and angry, healed up satisfactorily. In the same journal Dr. H. P. Taylor describes the treatment of a rodent ulcer of large size—one inch long and half-an-inch across. The treatment was not carried out very continuously, but the ulcer was reduced and formed a complete cure at the time of writing the paper.

### Further Probable Applications of "Ionic" Medication.

This treatment of rodent ulcer by zinc is really only the first fruits of what I hope may be a series of successes in electro therapeutic work. One can think of numerous conditions where some such treatment by a drug locally and thoroughly applied may be efficacious, as, for instance, in ringworm, by the introduction of an antiseptic. I have made experiments and have got suggestive results by introducing copper into a ringworm patch. This is sometimes followed at once by the disappearance of the ringworm and the development of the healthy hair. Copper introduced in that way, however, does destroy the fungus. I also learned that copper so introduced in larger quantities may provide a method of permanently destroying hair. The introduction of magnesium "ions" has startling effects in cases of multiple warts on the hands. It is possible that we may shortly be able to cure lupus by the introduction of some antiseptic. I have been working on this without much success hitherto. Lupus ought to be amenable to such treatment as this, but unfortunately the tubercle bacillus contains forty per cent. to fifty per cent. of fat—almost a complete non-conductor of electricity. The tubercle bacillus is, therefore, protected from the forcible introduction of the "ions." But by the use of such substances as fuchsine, aniline and bodies of that class I hope to get better results. The method seems to be a good one and one deserving of study. The effect of zinc on patches of pustular eczema is striking. Perhaps some such method might be useful in the treatment of malignant disease. But the limitations of this method are that the "ions" can be introduced only very slowly, and a prolonged application would be required to reach parts lying at great depth. Betton Massey has been doing good work on this subject for years. He has treated inoperable cancer by plunging metallic needles of amalgamated zinc and other metals deeply into the substance of cancerous tissues and continuing the current for two or more hours with the patient under chloroform. He has reported a number of cases which appear to be successful. For the present, however, and for us there is plenty of work to be done by this method in cases which are easy before we need attempt those cases which present supreme difficulties.

